# Undifferentiated small round cell sarcomas in the retroperitoneal space in a 12-year-old female: a rare case report

**DOI:** 10.3389/fped.2025.1602157

**Published:** 2025-08-29

**Authors:** Dan Liu, Xiaoge Liu, Yan Deng, Ran Wu, Xin Li

**Affiliations:** ^1^ Department of Radiology, Sichuan Provincial People’s Hospital, University of Electronic Science and Technology, Chengdu, China; ^2^ Department of Ultrasound, Ya’ an People’s Hospital, Ya’ an, China

**Keywords:** undifferentiated small round cell sarcoma, teratoma, retroperitoneal space, CT, *EWSR1* translocation

## Abstract

Undifferentiated small round cell sarcoma (USRCS) is an extremely rare and highly aggressive group of malignant neoplasms affecting bones and soft tissues. USRCSs, particularly extraosseous variants pose significant diagnostic challenges due to their rarity, similar clinicoradiological features, nonspecific morphology and the necessity for comprehensive molecular analyses. This paper discusses a rare interesting case of retroperitoneal USRCS in a 12-year-old female with a five-day history of diarrhea and slight right lower abdominal pain. Hematological profile, renal function test, liver function test and tumor markers were in normal limits. Preoperative imaging revealed a well-defined, highly vascularized mass in the lower right retroperitoneal space, featuring patchy calcification and osseous and fatty components that compressed the right ureter, causing obstructive hydroureteronephrosis. Given the clinical and imaging findings. The lesion was initially misdiagnosed as a benign teratoma. However, postoperative pathology and genetic testing confirmed USRCS, remarkably, the Ki-67 index was only 10%. The patient did not undergo any additional postoperative treatment and achieved long-term survival. Despite USRCS in the retroperitoneal space with calcification has been reported, we believe this is the first published case of USRCS with calcification and osseous and fatty components mimicking teratoma. The case is important in that it demonstrates the unusual imaging appearance of retroperitoneal USRCS.

## Introduction

Undifferentiated small round cell sarcoma(s) (USRCS) is a group of malignant tumors of uncertain histogenesis that predominantly arise in bones, but rarely in the soft tissues (1). Extraskeletal USRCSs have been found in the head and neck, skin, breast, lung, bone, and deep abdominal wall (2–5); however, USRCS in the retroperitoneal space is extremely rare (6–9). To date, less than 100 cases of retroperitoneal USRCS [including Ewing sarcoma (ES)/primitive neuroectodermal tumor (PNET)] have been published in the medical literature since it was first described in 1981 (10). In addition and, to the best of our knowledge, there have been no reports of retroperitoneal USRCS exhibiting calcification and osseous and fat production. Herein, we present a rare case of a malignant tumor located in the posterior abdominal cavity involving the right ureter. The pathological diagnosis was USRCS with calcification, and osseous and fatty components. This presented a diagnostic challenge because the imaging characteristics and intraoperative findings resembled a benign teratoma. We report imaging findings and discuss the pathogenesis and histogenesis of USRCS.

## Case report

**Figure 1:**
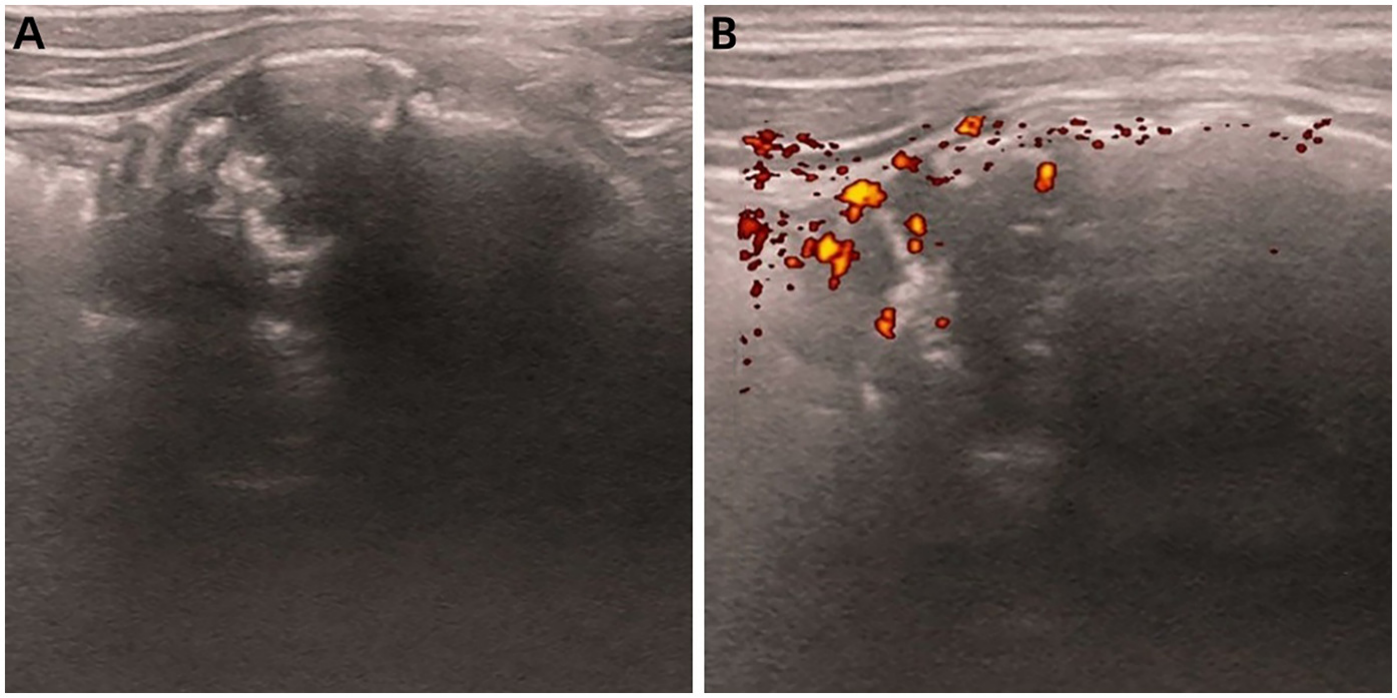
Abdominal ultrasound images of retroperitoneal USRCS in a 12-year-old girl. **(A)** Plain image shows a heterogeneous echoic mass with posterior acoustic shadowing in the right retroperitoneal region. **(B)** Transverse color Doppler image shows apparent flow within this structure.

**Figure 2:**
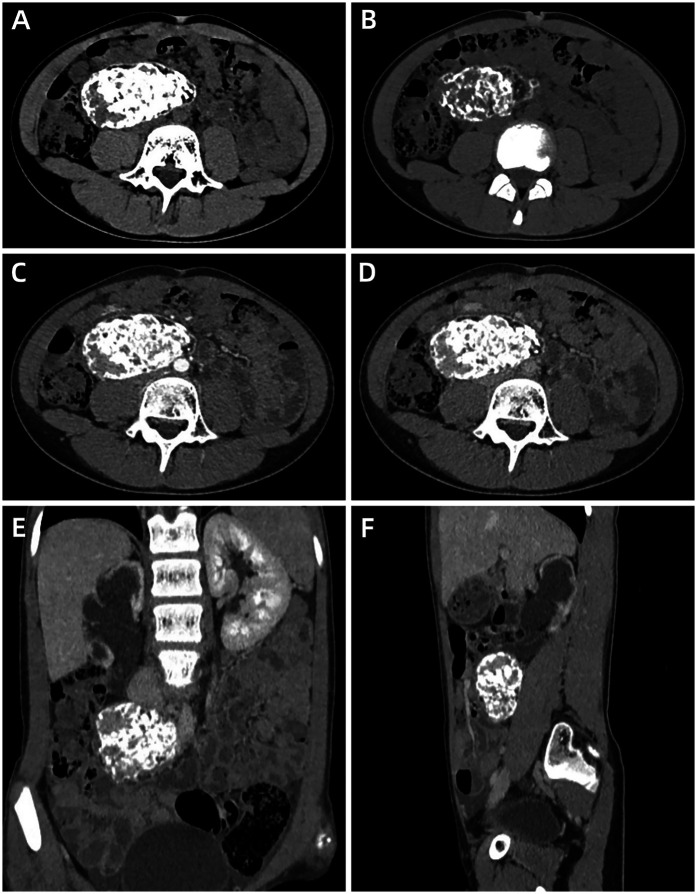
Abdominal contrast-enhanced computed tomography of a retroperitoneal undifferentiated small round cell sarcoma in a 12-year-old girl. **(A)** (soft window), **(B)** (bone window) Plain axial images show a mixed mass with areas of patchy calcification, osseous and fat components size in 7.3 cm × 6.6 cm × 4.0 cm, complete capsule. **(C,D)** Arterial phase **(C)** and portal venous phase **(D)** axial images show obvious enhancement of the lesion. **(E,F)** Coronal **(E)** and sagittal **(F)** images demonstrate that the lesion compressed the right ureter and caused obstructive hydroureteronephrosis.

**Figure 3:**
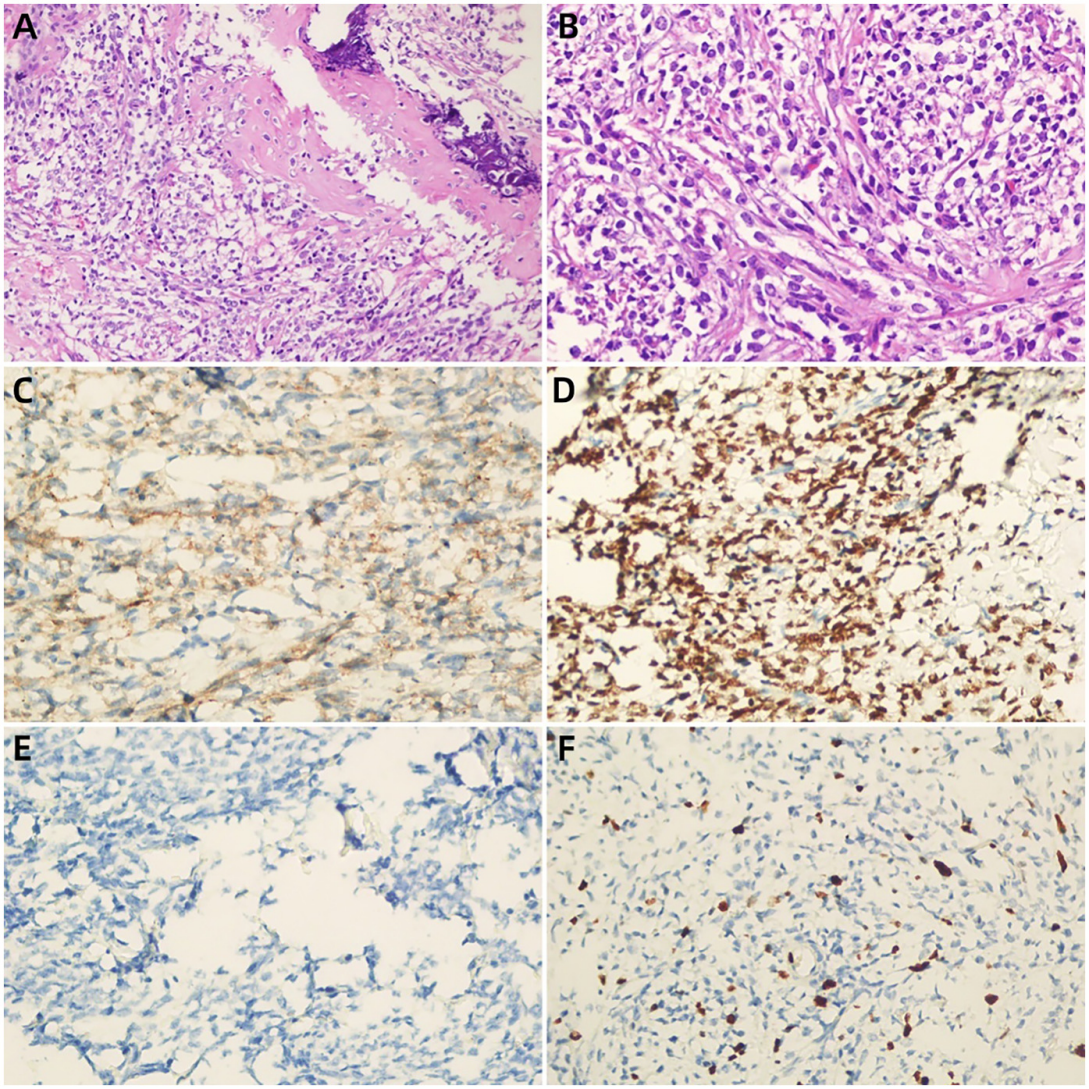
**(A,B)** (H&E) the tumor cells were small round with sparse cytoplasm, round nuclei and obscure nucleoli, suggesting undifferentiated small round cell sarcoma (a × 200, b × 400),areas of osseous matrix with calcification were observed;immunohistochemical findings showed strongly positivity for CD99 **(C)**, NKX2.2 **(D)** and negative for AFP **(E)** the Ki-67 labeling index was approximately 10% **(F)**.

A 12-year-old girl presented with a five-day history of diarrhea and slight right lower abdominal pain. Family history did not reveal any malignancies; in particular, there was no history of reproductive system tumors or rare tumors. Physical examination revealed a large, well-defined, palpable mass, measuring approximately 5 cm × 5 cm in the right lower quadrant, and routine laboratory investigations including creatinine, urea nitrogen, glomerular filtration rate, lactate dehydrogenase, and alkaline phosphatase levels were normal. Serum tumor markers, such as CEA, AFP, CA125, CA15-3, CA19-9, CA 72-4, ferritin (FER), CA 50, and the total human chorionic gonadotropin level were within normal limits. Abdominal ultrasonography (USG) (Figure 1) and contrast-enhanced computed tomography (CECT) (Figure 2) revealed a heterogeneous mass with areas of calcification, and osseous and fatty components, measuring 7.3 cm × 6.6 cm × 4.0 cm, in the right posterior abdominal cavity with a rich blood flow. The lesion compressed the right ureter, ileum, cecum, inferior vena cava, abdominal aorta, right common iliac artery, and the internal and external iliac arteries without definitive invasion, resulting in obstructive hydroureteronephrosis. The volume of the right kidney and blood perfusion were reduced. There was no evidence of regional or distant lymphovascular invasion. Based on clinical manifestations and imaging findings, the mass was suspected to be a teratoma. The patient was scheduled for surgery within 1 week of presentation. Upon exploration of the abdomen, a mobile retroperitoneal mass, measuring 7 cm × 6 cm × 5 cm, was identified, which was intimately adhered to the vessels, digestive tract and right ureter without invasion or encasement. The entire mass was removed without any complications or spillage. A ureteral stent was implanted under ureteroscopy, preserving the right ureter, and postoperative renal function and hydronephrosis significantly improved. On the cut surface, there was no hair or bone in the tumor. Microscopic examination revealed that the tumor was composed of small round and spindle-shaped cells with scarce cytoplasm and clear vesiculated nuclei that proliferated in sheets or solid nests with bands of hyalinized fibrous tissue. Areas of osseous matrix production with calcification were also observed; rosettes were not observed. Immunohistochemical examination revealed the following: positive expression of CD99, NKX2.2, and SATB2; negative expression of AFP, CD30, CD56, CK, Desmin, S-100, SALL4, SMA, CgA, Myogenin, p63 and Syn; and a low proliferation rate, as indicated by Ki-67 (10%) (Figure 3). Dual-color, break-apart probe fluorescence *in situ* hybridization revealed no *EWSR1* gene translocation and/or rearrangement. A search for other sites of tumor involvement using whole-body plain CT performed at an outside hospital revealed no abnormalities. The components of undifferentiated small round cell sarcoma in this case could not be further classified due to limited detection capability and economic limitations. These findings supported the diagnosis of localized USRCS arising from the posterior abdominal cavity. However, the patient refused adjuvant standard postoperative chemotherapy and/or radiotherapy. Abdominal USG was performed every 6 months at a local hospital. At the 25-month postoperative follow-up, the patient was alive without any signs of recurrence.

## Discussion

We report a rare case of retroperitoneal USRCS mimicking a teratoma, which, to our knowledge, has not been previously reported. This case is unique and interesting due to its unusual location, imaging findings, and biological behavior. Preoperative differential diagnosis of teratoma is difficult, which may cause delays in treatment. Pathological and IHC examinations, along with genetic testing, are crucial for accurate diagnosis and classification.

USRCS represents a heterogeneous group of small round cell tumors in the bone and soft tissues that share histological features but exhibit distinct molecular and clinical behaviors. The latest World Health Organization classification of soft tissue and bone tumors recognizes 4 categories within this group: ES, round cell sarcoma with EWSR1-non-ETS fusions, CIC-rearranged sarcoma, and sarcoma with BCOR alterations (11). ES is driven by fusions of EWSR1 (or much less frequently FUS) and ETS gene family members, most commonly FLI1 (approximately 90%) followed by ERG (approximately 5%) (12). Approximately 80%–90% of ES cases originate in the bones. The ribs, pelvis, or the diaphysis or metadiaphysis of long bones are most frequently involved. Approximately 10%–20% of ES occur in soft tissue, particularly in adults (13). CIC-rearranged sarcoma is defined by the presence of fusion involving CIC gene. Over 90% of tumors have CIC::DUX4 fusion (14), whereas rare alternative fusion partners include NUTM1, FOXO4, LEUTX, and NUTM2A (15). Most CIC-rearranged sarcomas present in the deep soft tissues of the head and neck, retroperitoneum, or pelvis. BCOR alteration sarcoma includes at least three molecular subcategories: BCOR::CCNB3 fusions, BCOR-alternative non-CCNB3 partner fusions and BCOR ITD (16). BCOR-rearranged sarcomas tend to occur more frequently in bone than in soft tissues, accounting for approximately 4% of round cell sarcomas. BCOR–CCNB3 sarcoma involves more frequently the pelvis, the lower limbs, and the paraspinal region. Round cell sarcoma with EWSR1-non-ETS fusions includes two distinct entities, the EWSR1::NFATc2- and the EWSR1::PATZ1-fusion sarcomas. NFATc2 is a transcription factor with an important role in immune regulation and thereby without any relationship to the ETS transcription factor family associated with ES (17). PATZ1 is also located on chromosome 22, only two megabases away from EWSR1, leading to an intrachromosomal rearrangement with pericentric inversion (18). The former occur more frequently in osseous locations rather than in the soft tissues (∼2:1). The latter has a strong predilection for the thoracoabdominal region. However, the aforementioned sites are not absolute; there is an overlap in their occurrence locations. Some cases of USRCS have been reported in the head and neck, lungs, breasts, kidneys, adrenal glands, gastrointestinal tract, and other visceral organs (2–5); however, cases occurring in the retroperitoneal space are extremely rare. Based on a literature review, less than 100 cases of primary USRCSs in the retroperitoneal space have been reported to date (6–9).

The radiological features of USRCS in the retroperitoneal space are nonspecific. On CT/MRI, USRCS usually appear as ill-defined, irregular, or lobular moderately heterogeneous masses with cystic and necrotic areas, and rarely as calcification and hemorrhage, as well as mild-to-moderate heterogeneous enhancement (6–9). Interestingly, in our case, the tumor was a well-defined, regular, mixed-density mass with patchy calcification, osseous components, and fat components, as revealed by CT and USG suggesting that the tumor was a teratoma, but did not raise the suspicion of sarcoma. Although, some researchers (19–21) reported ES/PNET in the retroperitoneal space that presented as lobulated masses with calcification or osseous components, in our case, we identified numerous patchy calcification, and osseous and fatty components accounting more than 80% of the tumor. To our knowledge, such findings have not been previously reported in the medical literature. We speculated that the heterogeneity of the imaging findings may be related to the origin of tumor cells. Although the developmental origins of USRCS have not been precisely determined, accumulating evidence over the past 2 decades suggests that USRCS may arise from mesenchymal stem cells (MSCs) or progenitor cells of specific differentiation stages (22). MSCs are a type of stem cell with multilineage differentiation potential and can differentiate into various human tissues, such as adipogenic, chondrogenic, and osteogenic tissues. In addition, we speculated that USRCS in the retroperitoneal space originated from the transformation of the teratoma along the mesodermal lines. Teratomas are pluripotent tissues capable of transforming into endodermal, ectodermal, or mesodermal lines. Subsequently, it can transform into a PNET, carcinoma, sarcoma, or carcinosarcoma (23). In our case, IHC results revealed negative expression of AFP, CK, S-100, SMA, Myogenin, Syn. As such, this possibility was excluded.

USRCS is a highly invasive and rapidly growing malignant tumor with poor prognosis. ES are highly aggressive with metastases detected at presentation in approximately one in four patients. Despite current aggressive cytotoxic treatment regimens the 5-year OS of patients with metastatic ES ranges from 20% to 35% (24). Even in primary nonmetastatic disease 30%–40% of patients experience recurrence, either local, distant or combined, during follow-up. Survival after recurrence is poor, with 5-year post-relapse survival varying from 15% to 25%, local recurrence doing better than distant recurrence (25). CIC-rearranged sarcomas are molecularly defined, aggressive tumors. With a 5-year overall survival of around 50%, the outcome is poor. There is a high potential for metastasis, often at the time of diagnosis (26).The prognosis of BCOR alteration sarcomas is similar to ES and much better than CIC-rearranged sarcomas (27). The prognosis data about the round cell sarcoma with EWSR1-non-ETS fusions is limited. Initial reports showed a very aggressive behavior (28), with high risk for metastatic spread to lung, pleura, soft tissues and lymph nodes. Although later studies described occasional cases that behaved in a more indolent fashion, however, with the caveat that follow-up of those studies was very short (29). Additional studies with longer follow-up will be essential to better understand their true biological potential.

Interestingly, in our case, the malignant tumor manifested benign behavior. First, on CT, a sarcoma was well-defined with a complete calcified capsule. Yi et al. reported (30) that 53.3% (8/15) of PNET in intra-abdominal and retroperitoneal regions had well-defined margins, which may have two reasons. First, abundant fat surrounding the mass created sharp contrast and a distinct natural boundary between the two different tissues. Second, the tumor's rapid expansion continuously compressed the surrounding connective tissues of the retroperitoneal space, resulting in a complete capsule. In our case, the lesion compressed the right ureter, causing obstructive hydroureteronephrosis. The volume and blood perfusion of the right kidney were reduced, while the volume of the left kidney was increased; however routine urianlysis was normal and there were no symptoms related to urinary tract obstruction. These findings suggest that the growth of the tumor was a chronic process. Second, there were numerous calcification and osseous and fatty components in the lesion mimicking a teratoma, suggesting benign behavior. Third, in our case, the Ki-67 index was only 10%, suggesting mitotic inactivity. In Yi et al. study, they found that the proliferation rate was relatively high (with a median Ki-67 index of 40%, ranging from 20% to 90%) in the 8 cases with clear margins. Given the rapid formation of the capsule, we hypothesized that the capsule did not have sufficient time to be destroyed by tumor invasion, even though its high malignancy and invasiveness.

Currently, there is no standard treatment for USRCS due to its global rarity. Most treatments involve early surgical resection and are supplemented with radiotherapy and chemotherapy. In the present case, the patient did not undergo any adjuvant treatment and is currently alive (25 months postoperatively) without any signs of recurrence. Further studies are needed to determine whether routine chemoradiotherapy is necessary for USRCS with benign radiological features and a low Ki-67 index.

## Conclusion

We report the first case of USRCS with calcification and osseous and fatty formation, arising from the right lower retroperitoneal space. Retroperitoneal USRCS can mimic teratoma and exhibit benign behaviors. Our report contributes to the clinical and imaging knowledge on this disease and increases awareness among clinicians and radiologists because early diagnosis of USRCS is imperative for management of the disease and improvement of prognosis.

## Data Availability

The original contributions presented in the study are included in the article/Supplementary Material, further inquiries can be directed to the corresponding author.
